# Insights into Kidney Dysplasia in Duplex Kidneys: From Radiologic Diagnosis to Histopathologic Understanding

**DOI:** 10.3390/biomedicines12051126

**Published:** 2024-05-18

**Authors:** Dominik Świętoń, Kamil Buczkowski, Piotr Czarniak, Andrzej Gołębiewski, Małgorzata Grzywińska, Mariusz J. Kujawa, Susan J. Back, Maciej Piskunowicz, Ewa Iżycka-Świeszewska

**Affiliations:** 1 2nd Department of Radiology, Medical University of Gdansk, 80-952 Gdansk, Poland; 2 Department of Pathology and Neuropathology, Medical University of Gdansk, 80-214 Gdansk, Poland; kamil-buczkowski@gumed.edu.pl (K.B.); ewa.izycka-swieszewska@gumed.edu.pl (E.I.-Ś.); 3 Department of Pathomorphology, Copernicus Hospitals, 80-803 Gdansk, Poland; 4 Department of Paediatrics, Nephrology and Hypertension, Medical University of Gdansk, 80-214 Gdansk, Poland; piotr.czarniak@gumed.edu.pl; 5 Department of Surgery and Urology for Children and Adolescents, Medical University of Gdansk, 80-214 Gdansk, Poland; 6 Neuroinformatics and Artificial Intelligence Laboratory, Department of Neurophysiology, Neuropsychology and Neuroinformatics, Medical University of Gdansk, 80-214 Gdansk, Poland; 7 Department of Radiology, Children’s Hospital of Philadelphia, Perelman School of Medicine, University of Pennsylvania, Philadelphia, PA 19104, USA; backs@email.chop.edu; 8 1st Department of Radiology, Medical University of Gdansk, 80-214 Gdansk, Poland

**Keywords:** kidney dysplasia, magnetic resonance urography, CAKUT

## Abstract

Duplex kidney is a urinary tract anomaly commonly associated with a wide range of primary and secondary parenchymal structural abnormalities. We present a unique comparison of US and MRI findings with histopathology following partial resection of duplex kidneys due to nephropathy. We examined a group of 21 children with duplex kidneys who were qualified for heminephrectomy (24 kidney units (KU)). All patients underwent US and MRI prior to the surgery. The imaging results were compared with histopathologic findings. In 21/24 KU, dysplastic changes were found on histopathology, including all with obstructive nephropathy and 7/10 specimens with refluxing uropathy. The loss of corticomedullary differentiation on US and increased signal on T2-weighted images (T2WI) on MRI were the imaging findings that best correlated with fibrosis. In children with megaureter, there were no statistical differences in histopathological findings between primary megaureter, megaureter with ureterocele, and megaureter with ectopia (*p* > 0.05). The extent of dysplasia of the affected pole correlated negatively with residual function in MRI. Kidney dysplasia and inflammation in the kidney with obstructive nephropathy are the most important histopathologic findings of this study. US is a valuable screening tool, and MRI enables morphologic and functional assessments of the nephropathy in duplex kidneys.

## 1. Introduction

Congenital anomalies of the kidneys and urinary tract (CAKUT) and congenital nephropathies are the most frequent causes of chronic kidney disease in children (48% and 10%, respectively) [1,2]. Of the groups, duplex kidney is one of the most common developmental variants, associated with a wide range of primary and secondary parenchymal disorders and disorganization, often characterized as kidney dysplasia [3].

Parenchymal disorders, including kidney dysplasia, are challenging diagnoses among children on the spectrum of CAKUT; however, recognizing them is important due to their influence on future renal function. With the increase in accessibility of routine pre- and postnatal ultrasound (US) and advances in imaging techniques like magnetic resonance imaging (MRI), children with CAKUT and kidney dysplasia are more frequently diagnosed at an earlier stage. Therefore, the histopathological term “dysplasia” has also been implemented into clinical and radiological terminology as kidney dysplasia or kidney dysplasia pattern [4].

The histopathologic findings in kidney dysplasia can vary, depending on the expression of the dysplasia and its origin. Dysplasia is characterized by a decrease in nephrons, the presence of persistent primitive ducts, and disorganized architecture of the renal parenchyma [5]. In some cases, cyst formation is observed that can be single or numerous and, in the most advanced cases, presents as polycystic kidney dysplasia. Other features, not necessary for the diagnosis, are the presence of metaplastic cartilage or bone (usually localized within the renal cortex), nodular renal blastema remnants, and proliferating unmyelinated nerve fibers [6]. The histopathological picture of kidney dysplasia is often overlapped by changes secondary to coexisting urinary outflow disorders—chronic inflammation, secondary fibrosis, glomerulosclerosis, and arteriolosclerosis [5]. The histopathological diagnosis of segmental dysplasia of the kidney is not problematic, but the focal form of dysplasia can be overlooked within the inflammatory and fibrotic process. Immunohistochemical staining with antibodies such as WT1, PAX2, PAX8, or BCL-2 can be used as a supportive tool [5].

As the histopathologic diagnosis of dysplasia is possible only after surgical resection or in autopsy material, imaging studies play a crucial role in in vivo diagnosis. Two imaging methods, US and MRI, are the most adequate for the diagnosis of uropathies during the neonatal and postnatal periods. US is a fundamental tool for screening and monitoring urinary tract anomalies but is also accepted for screening and monitoring kidney pathologies [7]. The sonographic features of kidney dysplasia are still debated depending on the type of dysplasia—primary, secondary (mainly classified as obstructive), or mixed—but not contested. The most described and most important features are increased parenchymal echogenicity and reduced or absent corticomedullary differentiation (CMD) with or without cortical thinning and the presence of parenchymal cysts [7,8]. Other features include decreased kidney length and volume. All these features are assessed during the imaging evaluation of the urinary tract and are used as surrogate markers of the histopathological diagnosis.

MRI is an advanced imaging technique that is radiation-free and provides unique information on urinary tract anatomy, renal function, and the severity of uropathy on functional MR urography. MRI has become an emerging tool for planning the treatment of complex urinary tract anomalies, given its high-quality morphologic and functional images and data [9,10,11]. The definition of kidney dysplasia in MRI seems to be more specific, based on decreased kidney size, increased signal in T2WI sequences, reduced CMD, and the presence of parenchymal cysts.

We selected a group of children with at least one duplex kidney who underwent heminephrectomy due to recurrent urinary tract infections, urine incontinence, an ectopic ureter, or an afunctional pole. The histopathologic, US, and MRI findings were analyzed with special attention paid to kidney dysplasia. To the best of our knowledge, this material presents a unique opportunity to compare imaging findings with histopathology in patients with segmental dysplasia in duplex kidneys.

## 2. Materials and Methods

Twenty-one children aged 6–216 months (mean: 28.8 months; median: 17 months; male: 8; females: 13; IQR: 13) were included in this retrospective study. A right or left heminephrectomy was performed in 18 children, while a bilateral heminephrectomy was performed in 3 children. The inclusion criterion was heminephrectomy of one pole of the duplex kidney due to suspected obstructive or nonobstructive uropathy. In all cases, US and MRI were performed prior to the surgery. The time interval between US and MRI was not more than 3 months.

A total of twenty-four renal specimens were analyzed. Each operated kidney was treated as a separate kidney unit (KU). The patients were qualified for surgery after multidisciplinary consultation (nephrologist, urologist, and radiologist). The lower pole was resected in seven cases, and the upper pole was resected in seventeen cases. Fourteen KUs were resected due to obstructive uropathy, while ten KUs were resected due to obstructive uropathy and vesicoureteral reflux (VUR).

The main indications for surgery were recurrent urinary tract infections, urine incontinence, an ectopic ureter, or an afunctional pole (Table 1).

### 2.1. Ultrasound

US examinations were performed by a pediatric nephrologist with 25 years of experience in renal US imaging. The exams were performed with a GE Voluson S8 (GE Medical Systems, Milwaukee, WI, USA) using linear, high-frequency probes 2–9 Mhz and 12–16 Mhz and a convex probe 1–6 Mhz.

In all children, US reports were retrospectively analyzed, and details regarding parenchymal echogenicity, CMD, parenchymal thinning, and presence of cysts were extracted (MRI Examination Analysis Section). The collecting system was assessed with special attention paid to the bladder and distal segments of the ureters and the presence of ureter ectopia, dysplastic constriction, or ureterocele.

### 2.2. MRI Examination Analysis

In all cases, MRI was performed, preceding surgery, in accordance with a standardized protocol on a Philips Achieva 3T TX magnetic resonance scanner (Philips Healthcare; Best, The Netherlands). The detailed analysis of the MRI images of the changed pole was performed and compared with the unresected pole and the contralateral kidney. The features analyzed included renal length, CMD, parenchymal signal on T2WI in comparison to the unchanged pole, and the presence of cystic changes. The collecting system was assessed, including the degree of dilatation and cause of uropathy, such as ureteropelvic junction obstruction, primary megaureter, ureteric ectopia, or ureterocele. In cases with megaureter, the distal ureter was assessed for the presence of ureterocele, ureteric ectopia, or a dysplastic fragment of the ureter.

Of the twenty-one children, seventeen underwent MRI with intra-venous contrast administration and function analysis; in five children, non-contrast MRI (MRI hydrography) was performed for anatomical assessment.

**Figure 1:**
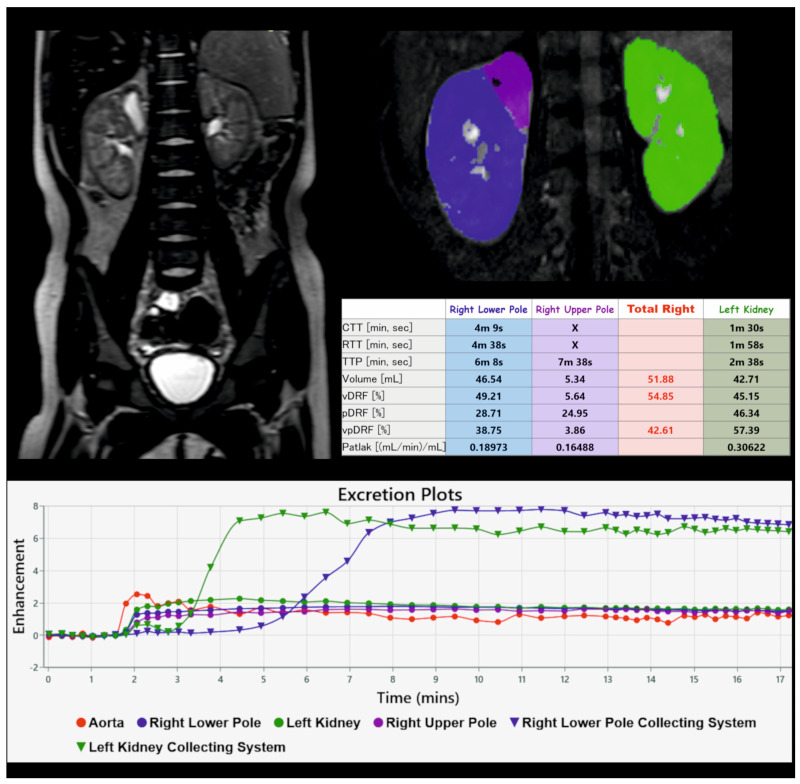
Example of kidney segmentation and functional analysis in ChopfMRU software.

The freeware, parametric magnetic resonance imaging v1.2.31-b (pMRI; www.parametricmri.com), was used for semiautomatic segmentation and calculation of renal function based on volume-enhanced parenchyma and the Patlak number [12]. The parameter vpDRF (volume Patlak differential renal function) was calculated. In kidneys with a duplex collecting system, vpDRF was calculated for each renal moiety separately (Figure 1).

### 2.3. Pathomorphological Analysis

The buffered, formalin-fixed heminephrectomy specimens were assessed macroscopically. The tissue samples were embedded in paraffin blocks using routine histotechnology procedures, and 4 µm slices were cut and stained with hematoxylin and eosin (H + E) and Masson’s trichrome (selected specimens). Microscopic evaluation was performed using an Olympus BX53 microscope (Olympus Corporation, Tokyo, Japan). The morphological assessment included dysplasia, parenchymal fibrosis, the severity of inflammatory changes, glomerulosclerosis, arteriolosclerosis, lymphoplasia, and microcalcifications.

**Figure 2:**
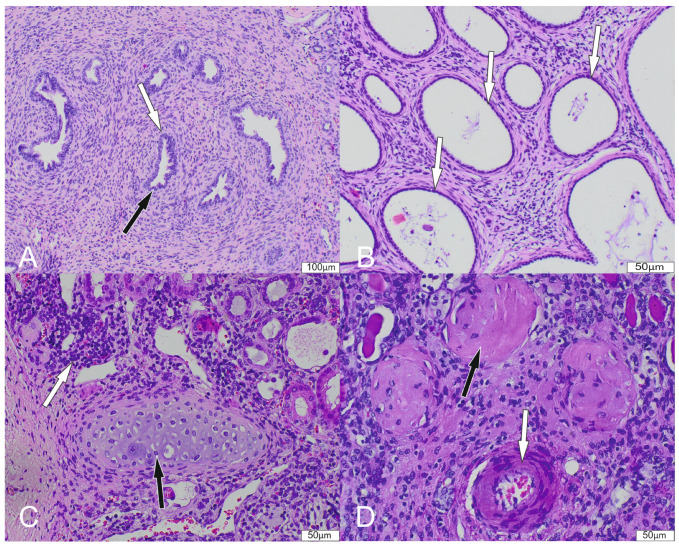
Histopathological findings in renal dysplasia. Histological slides stained with hematoxylin and eosin (H + E). The number in brackets indicates the corresponding magnification. (**A**) H + E (20×): Grouping of dysplastic tubules lined with a single layer of cuboidal and tall epithelium (black arrow) surrounded by fibromuscular collars (white arrow). (**B**) H + E (20×): Dysplastic kidney with formation of numerous small cysts (white arrow). (**C**) H + E (40×): A focus of metaplastic cartilage formation (black arrow) and areas of lymphocytic infiltration (white arrow). (**D**) H + E (40×): Thickened arterioles (white arrow) and numerous sclerosing glomeruli (black arrow).

The five criteria of renal dysplasia include the following, of which the first two are required for the diagnosis: (1) disorganization of the renal parenchyma, (2) presence of primitive nephron ducts lined with undifferentiated cuboidal/columnar epithelium and surrounded by a fibromuscular collar, (3) cyst formation, (4) metaplastic cartilage, and (5) irregular blood vessels. The extent of dysplasia was assessed as a percentage of involved surface of the sample on the H + E slides (Figure 2A–D).

Fibrosis was evaluated by estimating the percentage of the renal pole area remodeled by collagen fibers based on the H + E and Masson’s trichrome-stained slides. The percentage of sclerosed glomeruli was estimated in every specimen. The severities of chronic pyelonephritis and arteriolosclerosis were assessed using a semiquantitative scale (0—absent, 1—low, 2—moderate, 3—severe).

### 2.4. Statistics

Statistical analyses were performed using IBM SPSS Statistics v. 29.0.0.0 (IBM, Armonk, NY, USA). All results were checked for normal distribution, and the Shapiro–Wilk test was used to assess the normality of each parameter. If the test showed a non-normal distribution, the Spearman correlation test was used to demonstrate the correlation between the collected data. The independent-samples Kruskal–Wallis test was used to examine the difference between groups in dysplasia type and other parameters. Post hoc analysis was conducted to determine differences between the groups, and a significant difference was considered at *p* < 0.05.

## 3. Results

### 3.1. Ultrasound

**Figure 3:**
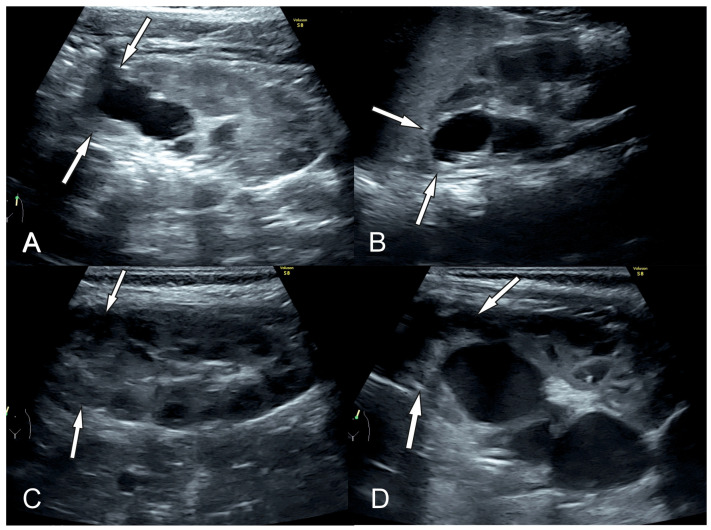
An example of ultrasound findings in two patients ((**A**,**B**) patient Nr 5R,5L; (**C**,**D**) patient Nr 12) with bilateral duplex kidneys with suspected segmental dysplasia. (**A**,**B**) Reduced parenchymal thickness and increased parenchymal echogenicity bilaterally (arrows). On the right, CMD of the upper pole is reduced (**A**) and absent in the left kidney (**B**). (**C**) The parenchymal thickness and CMD of the right kidney are preserved, inhomogeneous increased echogenicity of the upper pole. The right kidney was not operated. (**D**) The image presents reduced parenchymal thickness and increased parenchymal echogenicity of the upper pole in the left kidney; CMD is reduced.

On ultrasound, in all children (100%), a reduction in parenchymal thickness was observed (Figure 3). Increased parenchymal echogenicity was described in eighteen KUs, and normal echogenicity was described in six KUs. In four of the six KUs with normal echogenicity, the patients had a diagnosis of VUR, and all six patients had a history of UTI. CMD was preserved in four KUs, reduced in twelve cases, and absent in eight KUs (Table 2). We found a moderate correlation between CMD on US and histopathologic fibrosis (r = 0.508; *p* = 0.011), but no correlation was found between increased parenchymal echogenicity and histopathology (*p* > 0.05). Parenchymal cysts were observed in only two KUs (8% of cases). Urinary tract dilatation, if present, was diagnosed in US in all cases, and ureteral ectopia was diagnosed in 3/9 KUs (33%).

### 3.2. Magnetic Resonance Urography

**Figure 4:**
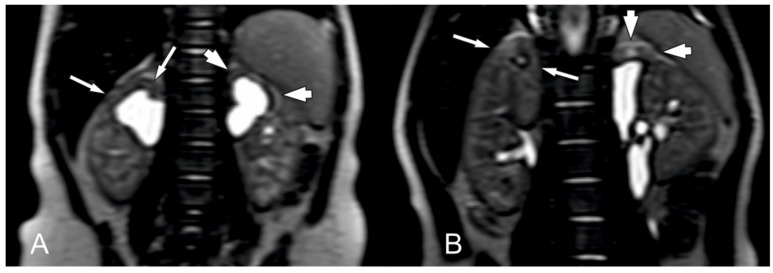
An example of MRI findings in T2-weighted image in two patients ((**A**) patient Nr 5R, 5L; (**B**) patient Nr 12) with bilateral duplex kidneys with suspected segmental dysplasia. (**A**) The parenchymal signal of upper poles in both kidneys is increased (lower extent in right kidney—long arrows), and parenchymal thickness and CMD are reduced (more advanced in left kidney—short arrows). (**B**) The parenchymal signal of upper pole of the right kidney is inhomogeneous and increased, parenchymal thickness is slightly thinner, and CMD is reduced (long arrows). The upper pole of the left kidney presents significantly reduced parenchymal thickness, increased parenchymal signal, and significantly reduced CMD (short arrows).

The most frequent finding in MRI was reduction in parenchymal thickness (100% of children), increased signal on T2WI of the affected parenchyma (21 KUs). The signal was decreased on T2WI in three KUs (Table 2) (Figure 4). There was a correlation between the changed signal on T2WI images and fibrosis in histopathology (r = 0.508, *p* = 0.011), not with the other histopathological findings (*p* > 0.05).

On MRI, complete loss of normal renal architecture was observed in two KUs. CMD was reduced in twenty KUs and preserved in two KUs. There was no significant correlation between CMD and histopathologic findings (*p* > 0.05), but histopathologic findings correlated with CMD on US examinations (r = 0.581, *p* = 0.003).

Cysts were found in nine KUs on MRI with single cysts in seven KUs and multi-cystic changes in two KUs. On histopathologic specimens, multi-cystic changes were described in three KUs as segmental dysplasia with multi-cystic changes; two of three correlated with MRI findings.

In the group of patients with MR urography, in 13/17 KUs, a residual urine (contrast) excretion was observed, and 4/17 were afunctional. A moderate negative correlation was found between reduced excretion of the contrasted urine and arteriosclerosis (r[15] = 0.612 m; *p* = 0.09) and with severity of fibrosis (r[15] = −0.595 m; *p* = 0.012) but not with the extent of dysplasia (*p* = 0.31).

The percentage of the remnant differential renal function of the affected pole was significantly lower in cases with histopathologically diagnosed dysplastic changes (*p* = 0.05). The mean DRF value of the resected pole was 8.9% (range 4–14%), while the DRF of the remnant pole of the operated kidney was 36.4% (range 19–47%). In all cases with afunctional renal poles, lymphoplasia with severe glomerulosclerosis and arterial fibrosis and microcalcifications were found (Table 2).

### 3.3. Histopathology

**Figure 5:**
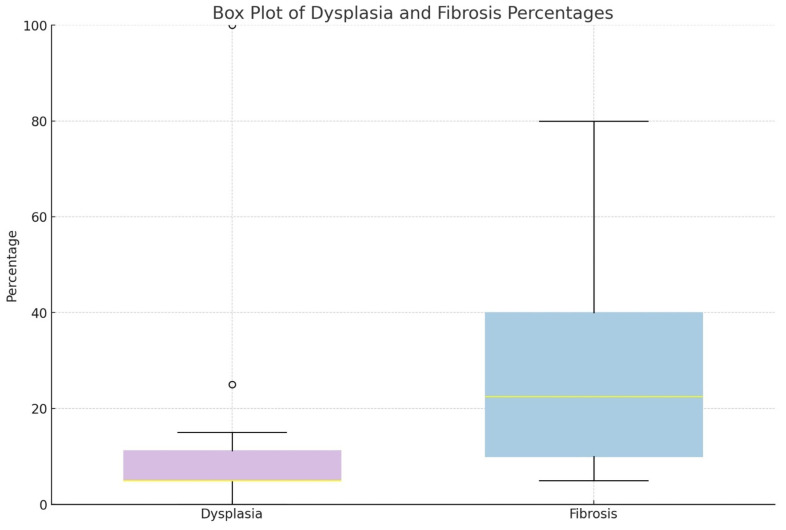
The box plot shows the distribution of dysplasia and fibrosis percentages from the patient data. Dysplasia percentages are represented alongside fibrosis percentages for a clear comparison.

Kidney dysplasia was diagnosed in 14/14 specimens with obstructive uropathy and seven out of ten cases with VUR. The distributions of dysplasia and fibrosis percentages in the analyzed samples are presented graphically (Figure 5), and the detailed results of histopathologic characteristics are presented in Table S1. The percentage of dysplasia in the examined samples was significantly higher in children with obstructive uropathy than in those with VUR (*p* = 0.007); the mean value of the percentage of dysplasia in the specimens was 17% and 7%, respectively. In three out of ten refluxing kidneys, no dysplastic changes were found in the microscopic analysis with fibrosis dominating in all cases up to 10% of the specimens along with chronic inflammatory changes. There was no difference in the extent of dysplasia between the children with or without UTI (*p* = 0.48).

Inflammation was found in all examined specimens independent of obstructive or refluxing nephropathy. In this group, sixteen children had a history of UTI, and eight children had no previous UTI. There was no statistically significant difference in the severity of inflammation between groups of children with or without UTI.

**Figure 6:**
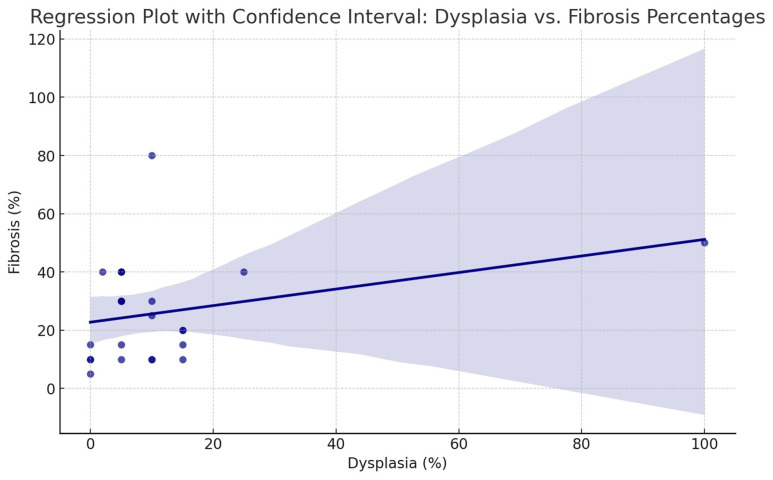
The regression plot illustrates the relationship between dysplasia and fibrosis percentages with a regression line that fits through the data points. The shaded area around the line represents the 95% confidence interval, indicating where we expect the true regression line to fall for the general population based on this sample.

Children with no dysplasia presented with less advanced fibrosis, a mean 10% of the specimen, while for children with dysplastic changes, the mean severity of fibrosis was 29% of the specimen (Figure 6). In children with megaureter, no significant histopathological differences were found between the group with primary megaureter, megaureter with ureterocele, and ectopic megaureter (*p* > 0.05).

## 4. Discussion

Dysplastic kidneys constitute a significant group of renal anomalies leading to chronic kidney disease [2]. Segmental dysplasia, a localized form limited to a part of the kidney, is commonly found in duplex kidneys. The changes often cause clinical dilemmas, particularly concerning the extent of surgical treatment required. Kidney dysplasia may accompany obstructive urinary tract anomalies like ureter ectopia or vesicoureteral reflux. As primary, non-obstructive kidney dysplasia can be diagnosed in radiologic studies supported with genetics, the diagnosis of secondary, mainly obstructive dysplasia is more demanding. Our study provides an opportunity to analyze histopathologic findings, radiological patterns, and clinical history in duplex kidneys.

The findings in US and MRI were complementary in most kidney characteristics. In US, increased parenchymal echogenicity with parenchymal thinning was observed in 18/24 KUs, presenting limited sensitivity for segmental kidney dysplasia. CMD feature did not correlate with the extent of dysplasia but correlated with fibrosis of the affected kidney. This important finding, indicating the loss of CMD, can be observed with time; simultaneously, the presence of CMD does not exclude kidney dysplasia. The US findings in obstructive nephropathy and dysplasia differ in frequency from primary, non-obstructive kidneys. Montini et al. studied, using a questionnaire survey, the knowledge and opinions about kidney dysplasia radiologic images among pediatric nephrologists. The most important and sensitive US feature of non-obstructive kidney dysplasia is parenchymal hyperechogenicity with reduced CMD [8]. Another valuable study is the meta-analysis conducted by Kohl et al. who concluded that reduced CMD with or without cortical thinning, independent of kidney size and the presence or absence of a cyst, is the most specific feature of non-obstructive kidney dysplasia in ultrasound [7]. Our study showed similar findings but with different proportions. According to our results, normal echogenicity and present CMD do not exclude kidney dysplasia and fibrosis. However, based on this study, we agree that, in most cases, increased echogenicity, thinning parenchyma, and reduced CMD are the most specific findings in obstructive kidney dysplasia. Interestingly, cysts are characterized with very low sensitivity in US.

The most frequent imaging findings in MRI were parenchymal thinning, changes in the T2WI signal of the parenchyma, and reduced CMD. CMD was preserved in only two cases; however, dysplasia was also found in the specimens. Despite the correlation of CMD in MRI with US, reduced or lost CMD seems to be a more specific and sensitive parameter in MRI than US. Interestingly, cystic changes were found in 36% of MRI exams and only 8% in US. The findings are consistent with previous studies, which describe the dysplastic pole as smaller with a significant reduction in parenchyma and CMD and, occasionally, the presence of small cysts [13]. In accordance with other authors, cysts in changed parenchyma in imaging studies are characteristic of kidney dysplasia but with low sensitivity [14]. US was equal with MRI in the assessment of urinary tract dilatation, diagnosing ureterocele, but insufficient in the visualization of ureter ectopia. This is in accordance with previous studies but is also an important finding for pediatric nephrologists and urologists [15].

We compared the differential renal function of the resected parts of the kidneys and the presence or absence of excretory function. We found a negative correlation between the degree of fibrosis, atherosclerosis, and dysplasia in the affected pole with present residual renal function, which is consistent with scintigraphy findings in previous studies [16]. The differential renal function of the resected poles was less than 14% with a mean of 9%. The correlation between the residual excretory function and extent of dysplasia forces precise assessment of the differential function of each pole. MRI has an advantage over scintigraphy in analyzing duplex kidneys because MRI provides better demarcation of the parenchyma for each pole, precise assessment of the differential renal function of each pole, and high-resolution anatomical images of the urinary tract [15,17].

The imaging findings of our study are based on a firmly selected group of patients; thus, the sensitivity and specificity of US and MRI findings in patients with less advanced nephropathies need further exploration. However, our findings establish the basis for further research in children with nephropathies and suspected kidney dysplasia.

A significant histopathologic result of our study is finding the difference between refluxing and non-refluxing renal poles. Dysplastic changes were more advanced in obstructive nephropathy than in reflux nephropathy and were even absent in three out of ten cases with VUR. This finding is generally in accordance with previous experimental studies, indicating the role of ureteral obstruction on dysplasia formation and inflammation [3,18]. Interestingly, we found no difference in inflammation in specimens in children with positive and negative histories of urinary tract infection. Also, there was no difference between inflammatory findings in histopathology in children with VUR and isolated obstructive nephropathy. This finding is important for clinical management, confirming the role of obstruction in stimulating the inflammatory process and, finally, leading to fibrosis, independent of urinary tract infections [19]. The results state a question about the treatment strategy in children with ureter obstruction, especially the conservative approach, as the fibrosis can progress with time, while dysplastic changes are restricted to the period of kidney development. Interestingly, there was no statistically significant difference in the extent of histologic findings, especially dysplasia, between kidneys with primary megaureter, megaureter with ureterocele, and megaureter with ectopia, which is consistent with other studies [20,21].

Summarizing the imaging findings, increased parenchymal echogenicity in US, increased signal in T2WI in MRI, reduced parenchymal thickness, and reduced CMD are the most common findings in obstructive nephropathy with dysplastic findings by histopathology. Preserved CMD or cyst absence does not exclude kidney dysplasia. US is a sensitive tool to assess changes in renal structure and urinary tract anomalies but seems to be insufficient in complex anomalies associated with duplex kidneys, especially due to low sensitivity in the diagnosis of ectopic ureter. MRI better characterizes urinary tract morphology and assesses differential renal function of each renal moiety, which enables the decision about further treatment. This is important since up to 50% of duplex kidneys are fraught with complications, like urinary tract dilation, calculi, urinary tract infection, dysuria, bladder dysfunction (ureterocele, ureteral ectopia), and need for surgical treatment [11,17,22,23,24].

### Limitations

The main limitation of this study is the small number of the analyzed group, due to the difficulties in collecting such a homogenous group of patients. The second limitation is the firmly selected group of patients with advanced nephropathies, which can influence the sensitivity and specificity of the imaging findings.

## 5. Conclusions

This study gives a unique insight into imaging findings in correlation with histopathology in duplex kidneys after heminephrectomy.

The findings confirm the relationship between urinary tract obstruction and kidney dysplasia as well as with the inflammatory process.

US is a valuable screening tool, and MRI is highly efficient in the morphologic and functional assessments of complex urinary tract anomalies, important in qualification for surgical treatment.

## Figures and Tables

**Table 1:** Clinical characterization of the examined group.

Patient Number	Age (months)	Resected Pole	Ectopic Ureter	Ureterocele	Vesicoureteral Reflux	Recurrent UTI	DRF of the Resected Pole *	DRF of the Remnant Pole of the Operated Kidney *
1	9	Right upper	No	Yes	Yes	Yes	12.0%	35.7%
2	10	Left upper	No	Yes	No	No	11.0%	30.0%
3	18	Left upper	Yes	No	No	No	n.d.	n.d.
4	20	Left lower	No	No	Yes	Yes	2.6%	37.0%
5R	13	Right upper	No	No	No	No	7.0%	44.7%
5L	13	Left upper	No	No	No	No	6.6%	41.0%
6	14	Left lower	No	No	Yes	Yes	10.0%	36.0%
7	11	Left upper	No	No	Yes	Yes	9.0%	33.3%
8	8	Right upper	Yes	No	No	Yes	5.5%	39.0%
9	24	Right lower	No	No	No	No	13.0%	19.3%
10	44	Right lower	No	No	Yes	No	n.d.	n.d.
11LL	57	Left lower	No	No	Yes	Yes	n.d.	n.d.
11LU	19	Left upper	No	Yes	No	Yes	n.d.	n.d.
12	22	Left upper	No	No	No	Yes	3.0%	39.0%
13	19	Left lower	No	No	Yes	Yes	n.d.	n.d.
14	20	Left upper	Yes	No	No	No	8.0%	47.0%
15	57	Left upper	Yes	No	No	No	n.d.	n.d.
16	216	Right upper	No	Yes	No	Yes	8.0%	39.0%
17	11	Left upper	Yes	No	No	Yes	5.5%	38.0%
18	16	Left lower	No	No	Yes	No	14.0%	27.0%
19	53	Right upper	Yes	No	Yes	Yes	3.9%	38.8%
20	7	Left upper	Yes	No	Yes	Yes	n.d.	n.d.
21R	6	Right upper	Yes	No	No	Yes	14.0%	36.5%
21L	6	Left upper	Yes	No	No	Yes	11.0%	38.0%

DRF—differential renal function in magnetic resonance urography, L—left kidney, R—right kidney, LU—left upper pole of the kidney, LL—left lower pole of the kidney, * n.d.—no data—functional sequence with contrast not performed.

**Table 2:** US and MRI findings of the resected renal poles in the examined group.

Initials	Increased T2WI Signal of Renal Parenchyma in MRI	Increased Echogenicity of Renal Parenchyma in US	CMD *		Cysts		Residual Renal Urine Excretion in the Affected Pole in MRI **
			MRI	US	MRI	US	
1	Yes	Yes	1	0	No	No	No
2	Yes	Yes	1	1	Yes	No	Yes
3	Yes	Yes	1	1	No	No	n.d.
4	Yes	No	1	2	No	No	Yes
5R	Yes	Yes	1	1	Yes	No	No
5L	Yes	Yes	1	0	No	No	Yes
6	Yes	Yes	0	0	No	No	Yes
7	No	Yes	1	1	Yes	No	Yes
8	Yes	No	1	1	Yes	No	No
9	Yes	Yes	1	0	No	No	Yes
10	Yes	Yes	1	2	No	No	n.d.
11LL	No	Yes	1	1	No	No	n.d.
11LU	No	Yes	0	0	Yes	Yes	n.d.
12	Yes	Yes	1	1	Yes	No	Yes
13	Yes	No	1	1	Yes	No	n.d.
14	Yes	Yes	1	0	No	No	Yes
15	Yes	Yes	1	0	Yes	Yes	n.d.
16	Yes	Yes	1	0	No	No	Yes
17	Yes	No	1	1	Yes	No	Yes
18	Yes	Yes	1	1	No	No	Yes
19	Yes	No	1	1	No	No	No
20	Yes	No	1	1	No	No	n.d.
21R	Yes	Yes	2	2	No	No	Yes
21L	Yes	Yes	2	2	No	No	Yes

L—left kidney, R—right kidney, LU—left upper pole of the kidney, LL—left lower pole of the kidney, * 0—absent, 1—reduced, 2—normal, ** n.d.—no data—functional sequence with contrast not performed.

## Data Availability

Data are contained within the article and Supplementary Materials.

## Supplementary Materials

The following supporting information can be downloaded at: https://www.mdpi.com/article/10.3390/biomedicines12051126/s1, Table S1. Histopathologic results in resected moieties in the examined group after heminephrectomy.
