# Supplementary material for: Insights into Kidney Dysplasia in Duplex Kidneys: From Radiologic Diagnosis to Histopathologic Understanding
Source: Biomedicines. 2024 May 18;12(5):1126. doi: 10.3390/biomedicines12051126 (PMC11117610; doi:10.3390/biomedicines12051126)
Supplement: Supplementary file 1 [file biomedicines-12-01126-s001.zip › biomedicines-2998463-supplementary.pdf]

Table S1. Histopathologic results in resected moieties in the examined group after heminephrectomy.

Patient number	Renal dysplasia	% of the dysplasia *	% of fibrosis **	The severity of inflammation***	Lymphoplasia	Arteriolosclerosis ****	Microcalcifications	Other
**1**	Yes	10%	80%	2	Yes	2	Yes	Cartilage formation foci
**2**	Yes	10%	10%	1	Yes	1	No	Cartilage formation foci
**3**	Yes	5%	40%	3	Yes	2	Yes	Tubular thyroidization
**4**	No	0%	10%	1	No	1	No	
**5R**	Yes	5%	30%	3	Yes	3	Yes	Tubular thyroidization
**5L**	Yes	5%	30%	3	Yes	2	Yes	Tubular thyroidization
**6**	Yes	15%	20%	2	Yes	1	Yes	
**7**	Yes	5%	10%	2	Yes	1	Yes	
**8**	Yes	10%	30%	3	Yes	2	Yes	Patchy suppurative inflammation
**9**	Yes	5%	40%	1	Yes	1	No	
**10**	No	0%	15%	1	Yes	1	No	
**11LL**	Yes	2%	40%	2	Yes	2	Yes	Vascular malformation in cortex
**11LU**	multicystic renal dysplasia	100%	50%	2	Yes	2	No	
**12**	multicystic renal dysplasia	25%	40%	2	Yes	2	Yes	
**13**	Yes	5%	15%	2	Yes	1	No	Cartilage formation foci
**14**	multicystic renal dysplasia	10%	25%	3	Yes	2	Yes	Tubular thyroidization
**15**	Yes	10%	10%	2	Yes	2	Yes	

**16**	Yes	5%	30%	2	Yes	1	No	
**17**	Yes	15%	20%	2	Yes	2	Yes	
**18**	No	0%	5%	1	Yes	1	No	
**19**	Yes	5%	40%	2	Yes	2	Yes	Subcapsular tortuous blood vessels
**20**	no dysplasia	0%	10%	2	Yes	1	Yes	
**21R**	Yes	15%	10%	3	Yes	1	No	
**21L**	Yes	15%	15%	3	Yes	2	No	

* The percentage of dysplasia in a specimen estimated on haematoxylin and eosin stained slides. ** The percentage of sclerosed glomeruli was estimated in every specimen. *** The degree of inflammation: 1 - low, 2 - moderate, 3 – severe ****The degree of arteriolosclerosis: 1 - low, 2 - moderate, 3 - severe

L – left kidney, R – Right kidney, LU – left upper pole of the kidney, LL – left lower pole of the kidney,
